# Correction: Crisis of confidence averted: Impairment of exercise economy and performance in elite race walkers by ketogenic low carbohydrate, high fat (LCHF) diet is reproducible

**DOI:** 10.1371/journal.pone.0235592

**Published:** 2020-06-26

**Authors:** Louise M. Burke, Avish P. Sharma, Ida A. Heikura, Sara F. Forbes, Melissa Holloway, Alannah K. A. McKay, Julia L. Bone, Jill J. Leckey, Marijke Welvaert, Megan L. Ross

There are symbols and a legend missing from Fig 5. Please see the correct Fig 5 here.

**Fig 5:**
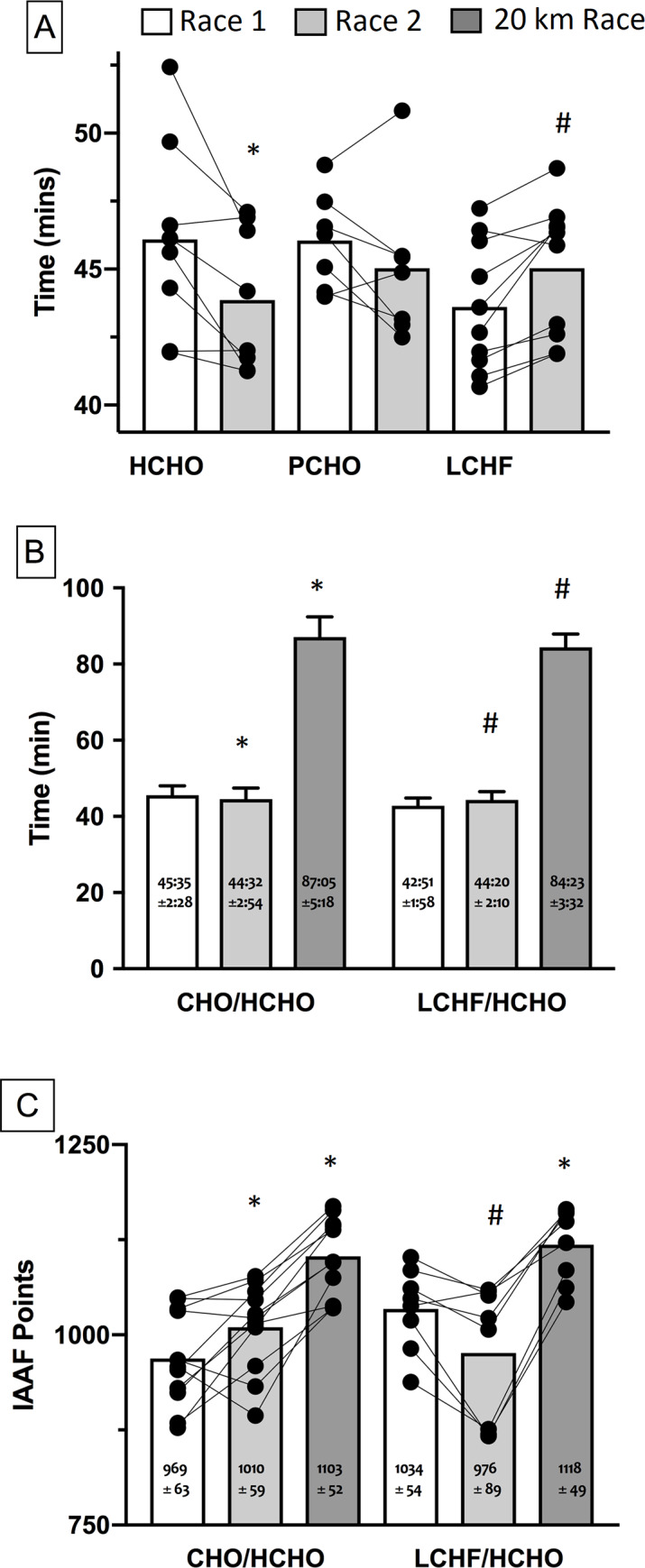
Race times for IAAF sanctioned events completed during study. A: 10,000 m race walk events in elite race walkers undertaken pre- (Race 1; Baseline) and post- (Race 2; Adapt) 25 d of intensified training and high carbohydrate availability (HCHO, n = 8); periodised carbohydrate availability (PCHO, n = 7) or ketogenic low carbohydrate high fat (LCHF, n = 10) diets. B: comparison of 10,000 m and 20 km race times for subgroup of participants who undertook 3 wk of de-adapt to HCHO diet and race taper after 25 d intensified training and HCHO or PCHO diet (n = 11) or LCHF diet (n = 8) and C: comparison of 10,000 and 20 km race outcomes of this subgroup expressed as IAAF ranking points. * = significantly faster than Race 1 (p< 0.01); # = significantly slower than Race 1 (p< 0.01).
