# Supplementary material for: Crisis of confidence averted: Impairment of exercise economy and performance in elite race walkers by ketogenic low carbohydrate, high fat (LCHF) diet is reproducible
Source: PLoS One. 2020 Jun 4;15(6):e0234027. doi: 10.1371/journal.pone.0234027 (PMC7272074; doi:10.1371/journal.pone.0234027)
Supplement: Attachment — Submitted filename: Burke et al (2019) PLoS One review rnd 1.docx [file pone.0234027.s002.docx]

**OVERALL**

This manuscript repeated a study previously done by the same group of authors. It assessed how different dietary programs, i.e. ketogenic, high carbohydrate and periodized carbohydrate, affected exercise economy and performance responses to a period of intensified exercise training. Although the dataset is largely a repeat of previous work, the authors addressed some criticisms of the initial manuscript in the current design, most notably adding a period of high carbohydrate availability between the end of intensified training and a race. The importance of repeatable data and the novel aspects to this study make this manuscript is deserving of publication in *PLOS One*, but some concerns must be addressed first.

**MAJOR REVISIONS**

**[1]** P-value reporting is challenging to interpret at times and sometimes p-values are not reported. Please include a p-value for each comparison mentioned even if not significant.

**[2]** Statistical analysis – reporting and method selection.

a. First, can you please explain your statistical analyses in greater detail here? To my understanding, a general linear mixed model is commonly used to predict something based on fixed and random effects. Due to this, I’m unsure how this would detect differences in time trial duration. It makes sense to me that you have entered diet and base/adapt as fixed effects and subject as random, but it seems like these would predict an outcome variable instead of test for differences between groups. Additionally, please justify why you feel this methodology is appropriate despite the relatively small sample size used.
b. Second, why did you select this method as opposed to an ANCOVA, where the covariate is subject training status or baseline time trial duration? I like that you are trying to control for baseline differences between subjects, however an ANCOVA may be a more appropriate method to achieve what you are trying to when considering that mixed models are typically used for very large sample sizes. It may also increase your chances of finding a difference between diet interventions at *Adapt*.
c. Third, when building this model why not include sex as a fixed factor? You have acknowledged it may affect you results but don’t include it in the model. Also, please list all fixed and random effects initially entered into the model and then which you decided to include and exclude. If you decide to keep with this analysis it is important to include this information for repeatability purposes.
d. Last, please include effect sizes for the physiological and performance effects. At the very least, it would be useful to add a Cohen’s dz or d for comparisons between two groups

**[3]** Graphical representation of data. Showing individual data points on your graph, or at least plots that show more data than mean + SD, are recommended. It would be nice to see individual data points that are connected for repeated measures, i.e. Figure 3 showing a line between *Baseline* and *Adapt* within each diet that connects the two points for each subject. Although significant statistically it is still informative, especially considering the relatively small N, to show the individual responses. I feel that this is particularly important for Figures 3 and 5 (economy and performance data), and less important and likely not necessary for metabolite data.

**[4]** There is a lot of writing throughout that seems to not be essential for a scientific audience, particularly in the discussion. For example, explanations of scientific processes (i.e. L669-674). Please consider condensing. Likewise, the nitrate-nitrite-NO reduction pathway discussion and relevance to BRJ responses in the discussion, although somewhat relevant, do not directly relate to the data set. The authors should consider only elaborating on points that directly relate to their dataset, and it is recommended that they spend more time talking about PCHO results instead.

**MINOR REVISIONS**

**Abstract**

L51. Consider another phase instead of keto-adaptation. Adaptation refers to changes over generations within a species; and therefore, adaptations would not have occurred in this study. Please revise throughout or provide an explanation as to why this is the most appropriate descriptor.

**Introduction**

The introduction was good for LCHF and HCHO groups, however it did not effectively introduce the PCHO group. Please include some more background on and rationale for having a PCHO group.

**Methods**

Were any of the subjects who participated in this study also in your previous study? Particularly anyone in the LCHF group. Considering you are trying to replicate results, it is important for the reader to know if this is a completely, or partially, different subject group.

L167. Is there a more appropriate word than “succumbed” to describe what happened?

L299. Do you have any data to prove that athletes are indeed at steady state during this time?

L306. Did you take metabolite measurements in single, duplicate, triplicate? Please specify.

L306. The analyzer you used measures D-b-hydroxybutyrate only. Since, it does not measure other ketone bodies (acetone and acetoacetate) it is incorrect to say “ketones”. Please change the label to beta-hydroxybutyrate throughout.

L309. This is a good control method. Do you have any data/evidence to support inter-device variability?

L313. Please specify that these are estimated rates of “whole-body” CHO and fat oxidation.

L318-323. Regarding not including ketone oxidation to VO2 and RER. I agree that you should not have ketone body oxidation in your calculations because you don’t have the data to calculate it without making significant stretches. Furthermore, some of your “ketone” data show it increases pre- to post-exercise (L520); and therefore, if using the same calculations as Cox et al. 2016 *Cell Metab*, you would get a negative contribution of “ketone bodies” to VO2. My concern, however, is the error from not including this is not necessarily systematic because you have a training program and exercise training has been implicated with an increased ability of skeletal muscle to oxidize ketone bodies (For review see Evans et al. 2017, *J Phys*). Thus, the error obtained from excluding ketone bodies in your energy expenditure estimations may be larger post- vs. pre-training.

L348. Why not have everyone consume a HCHO pre-race? Based on the differences you found when comparing *Base* vs. *Adapt* within diet treatments and the non-significant difference in 20 km TT performance at the end, the data set cannot distinguish whether the impaired performance on LCHF was due to a single LCHF meal pre-race or chronic LCHF intake during training. Also, when comparing LCHF at *Adapt* to *Baseline*, the pre-race meals would have been different because you said all were on a HCHO diet at *Baseline*. This should receive more attention in the discussion. Please advise if I have misunderstood the pre-race diet data.

Power calculation. You performed an *a priori* power calculation, however, did not include enough data to make it repeatable. Please include the effect size, alpha, power, statistical test, etc. that were input into your calculation and state the outcome variable the calculation was performed on.

L372. Can you clarify what you mean by “random intercept for the athletes”. Do you mean a certain characteristic of athletes, i.e. aerobic fitness or time-trial (TT) duration at *Baseline*, or is it simply a random intercept for “subject”? Also, would it be better to include *Baseline* or personal best TT duration when assessing differences in TT data at *Adapt* instead of aerobic fitness?

It is good you checked normality with a QQ plot, however the authors are recommended to also include an objective normality test to compliment this subjective normality test.

L382. Why did you use Bonferroni *post-hoc* test as opposed to the more conventional Tukey’s HSD?

L384. Please report which variables did not pass the normality test.

How was normality assessed for diet intake data? This should be added to the manuscript.

L385. When analyzing diet, you ignored macronutrient composition in your ANOVA/t-test. I.e., to my understanding, diet was analyzed with a t-test (group) within a macronutrient vs. 2-way ANOVA (group x macronutrient). Does analyzing the data this way change the story?

L543. How did you normalize these data? I.e. relative to race 1 performance?

Please explain why effect sizes were not computed for this study, particularly for performance and economy data.

**Results**

Do you have Wpeak data? It would be interesting to see if LCHF had a higher Wpeak in concert with a higher VO2peak, or if the higher VO2peak was simply a result of reduced BM and/or increased fat oxidation.

L419. Specify what groups each value belongs to.

L450. Specify if these changes were significant in addition to being “minor”.

L454. Did the comparison of VO2 significantly differ between diets at *Adapt*?

L463-468. Why is your economy data shown as relative VO2? The LCHF diet group lost BM and therefore irrespective of changes in economy would report higher relative VO2s than the other two groups regardless of changes in economy. It would be best to show this data as absolute VO2, or even better, use BM as a covariate in your analysis.

L583. The fact 3 participants got a personal best in LCHF/HCHO makes for an interesting talking point. Please add this to the discussion.

**Discussion**

Overall the discussion spent a lot of time on data that you had already shown in Burke et al. 2017. I recommend that you shorten this section as what is being discussed isn’t novel per se. It is more important to talk about, as you have highlighted in the intro, how it was repeatable.

L556. Do you have a reference to demonstrate that the intensities you assessed are similar to those that an ultra-endurance athlete competes at?

L656. The way this is worded makes it seem like HCHO and PCHO diets improved performance and LCHF decreased performance, although this is not that you found. Please consider rewording to better reflect that PCHO saw no change in performance.

L638. Do you have any insight into why blood ketones did not increase as much in this study (Figure 3, ~0.8 mM) vs. your previous work (~1.5 mM at same point)? Looking at absolute values there appears to be a slight difference in means.

L641. Some may argue that glycolytic activity was not the same despite similar blood lactates. A LCHF diet reduced PDH flux and transformation was more in an “inactive” state, which you mention later. Therefore, for the same pyruvate generation in LCHF vs. HCHO, you’d expect more lactate production in HCHO. Also lactate clearance from blood or muscle could have been affected, or lactate generation; or glycogenolysis could have been reduced. The authors go into more detail later, but should list some alternate explanations for blood lactate results in addition to the speculation they have proposed.

L661. Similar to my comment on ketone oxidation and training above, this error may not be systematic. Please address this.

L666. Including an effect size would help objectively make your point of a small but critical change.

L694. Including individual data or an effect size such as Cohen’s dz would make this point significantly stronger. Also, having ES for this study would make comparisons to your previous work stronger.

708. Did you account for differences in training quality and volume in your statistical model? How do we know the changes in TT performance aren’t because of differences in training volume reported in the first half of the program?

L734. Can you list any more muscle-specific consequences of this in addition to BP? The discussion mentions many muscle-specific effects of LCHF and including a muscle-specific effect here would complement the discussion nicely.

L775. Please clarify what you mean by and how you “*removed* the independent…”. Do you mean these were included in your statistical model?

L782. You should consider referencing Evans et al. 2018 and 2019 in MSSE in addition to Leckey et al. to make your argument stronger. These papers do not show an improvement in performance despite using the same supplement as ref 71 (Cox et al.), which showed a performance improvement.

L792-798. Similar to my main discussion comment this is a part of the discussion that seems unnecessary for a scientific audience and could be more concisely summarized by citing a review. Please consider shortening.

L808. Reference 78 is not in the reference list.

L832. Instead of chronic, consider stating duration in weeks as some people would argue that your exposure to LCHF was not “chronic”.

**Tables and Figures**

Figures 3 and 5. Please consider using individual data (as per above).

Figure 4. Change y-axis on 3C from “ketones” to “D-beta-hydroxybutyrate”.

Figure 5 B and C. Is there a graphical method to represent what you have described in your results section for this (L542-552). This is a really interesting statement and this method of analysis teases out a difference in a real-world time trial between diets. Also, please consider placing the 20 km race means for both diets beside each other to allow for easier comparisons. Additionally, if you are going to show individual data points here, it may be useful to assign each subject a symbol throughout to best show your repeated measures data because lines connecting 3 time points gets messy.
