# Supplementary material for: Crisis of confidence averted: Impairment of exercise economy and performance in elite race walkers by ketogenic low carbohydrate, high fat (LCHF) diet is reproducible
Source: PLoS One. 2020 Jun 4;15(6):e0234027. doi: 10.1371/journal.pone.0234027 (PMC7272074; doi:10.1371/journal.pone.0234027)
Supplement: Attachment — Submitted filename: response to reviewer.docx [file pone.0234027.s004.docx]

Thank you for the kind feedback on our revisions. We have addressed the remaining remarks as well as possible

Abstract

Introductory statement. Please consider adding something like “as compared to high and periodized carbohydrate diets.” at the end of your statement to better convey the study purpose.

Line 39. Merely a suggestion, but if you have words available it would help for clarity if you write “After Adapt, athletes resumed HCHO…”.

Line 43-44. Can you add a comparative statement about the trends for HCHO and PCHO groups for whole-body fat oxidation and oxygen cost?

Please have some mention of “rebound” performance for HCHO and PCHO groups in results.

L51. Please change “are robust” to “were repeated”.

We only had 12 words to enable us to make any changes in the abstract. We used these to better clarify the introductory statement, make line 39 clearer and to make the change to L 51.


Introduction

This reads very well. Thank you for implementing my suggestions.


Methods

All initial queries were sufficiently addressed.


Results

L418. This is a really cool finding and I agree with the suspected muscle glycogen and water content changes! Entirely out of curiosity, has any study directly measured this?

Thanks for the feedback. We have a separate paper that covers the changes in body composition and some health parameters (lipids, insulin sensitivity) so we will cover that aspect more clearly in that paper.

L429. Please ensure that p=0.987 and not p=0.0987

Yes, it is p = 0.987

How did you calculate a 95% CI for your effect size? To my knowledge Cohen’s d is the difference in means / SD of the differences and therefore wouldn’t give a 95% CI unless you had an effect size for each subject.
Also, regarding effect sizes, it will be more valuable to report Cohen’s dz as it better represents data in repeated-measure designs vs Cohen’s d. You have many small effect sizes that may be underrepresented compared to Cohen’s dz. Please consider revising.

We agree that a standard Cohen's d as you describe will not do the repeated measures design justice. However, since we used a linear mixed model estimates (i.e. accounting for the repeated measures) we can calculate a Cohen’s dby including the random effects in the denominator of the effect size (see methods). We have ensured that all are now calculated in this way, and that this has been explained in the methods section;

“Effect sizes based on the classical Cohen’s d were calculated from the linear mixed model estimates, while accounting for the study design by using the square root of the sum of all the variance components (specified random effects and residual error) in the denominator.”

L449. Please review p value that currently reads p>1.000. Revise all p-values to >0.99 at highest.

This has been done

Discussion

L839. Please revise “chronic”.

We have changed this to 3.5 wk

For conclusion, I suggest making the summary of your current findings past tense.

This has been done
